# The Impact of Biomaterial Cell Contact on the Immunopeptidome

**DOI:** 10.3389/fbioe.2020.571294

**Published:** 2020-12-16

**Authors:** Michael Ghosh, Hanna Hartmann, Meike Jakobi, Léo März, Leon Bichmann, Lena K. Freudenmann, Lena Mühlenbruch, Sören Segan, Hans-Georg Rammensee, Nicole Schneiderhan-Marra, Christopher Shipp, Stefan Stevanović, Thomas O. Joos

**Affiliations:** ^1^NMI Natural and Medical Sciences Institute at the University of Tübingen, Reutlingen, Germany; ^2^Department of Immunology, Interfaculty Institute for Cell Biology, University of Tübingen, Tübingen, Germany; ^3^Applied Bioinformatics, Center for Bioinformatics, University of Tübingen, Tübingen, Germany; ^4^DKFZ Partner Site Tübingen, German Cancer Consortium (DKTK), Tübingen, Germany; ^5^Cluster of Excellence iFIT (EXC 2180) “Image-Guided and Functionally Instructed Tumor Therapies”, University of Tübingen, Tübingen, Germany

**Keywords:** biocompatibility, monocytes, foreign body response, immunopeptidomics, mass spectrometry, host response, biomaterials, biocompatibility assay

## Abstract

Biomaterials play an increasing role in clinical applications and regenerative medicine. A perfectly designed biomaterial should restore the function of damaged tissue without triggering an undesirable immune response, initiate self-regeneration of the surrounding tissue and gradually degrade after implantation. The immune system is well recognized to play a major role in influencing the biocompatibility of implanted medical devices. To obtain a better understanding of the effects of biomaterials on the immune response, we have developed a highly sensitive novel test system capable of examining changes in the immune system by biomaterial. Here, we evaluated for the first time the immunopeptidome, a highly sensitive system that reflects cancer transformation, virus or drug influences and passes these cellular changes directly to T cells, as a test system to examine the effects of contact with materials. Since monocytes are one of the first immune cells reacting to biomaterials, we have tested the influence of different materials on the immunopeptidome of the monocytic THP-1 cell line. The tested materials included stainless steel, aluminum, zinc, high-density polyethylene, polyurethane films containing zinc diethyldithiocarbamate, copper, and zinc sulfate. The incubation with all material types resulted in significantly modulated peptides in the immunopeptidome, which were material-associated. The magnitude of induced changes in the immunopeptidome after the stimulation appeared comparable to that of bacterial lipopolysaccharides (LPS). The source proteins of many detected peptides are associated with cytotoxicity, fibrosis, autoimmunity, inflammation, and cellular stress. Considering all tested materials, it was found that the LPS-induced cytotoxicity-, inflammation- and cellular stress-associated HLA class I peptides were mainly induced by aluminum, whereas HLA class II peptides were mainly induced by stainless steel. These findings provide the first insights into the effects of biomaterials on the immunopeptidome. A more thorough understanding of these effects may enable the design of more biocompatible implant materials using *in vitro* models in future. Such efforts will provide a deeper understanding of possible immune responses induced by biomaterials such as fibrosis, inflammation, cytotoxicity, and autoimmune reactions.

## Introduction

The immunopeptidome is a sensitive composition of manifold HLA-presented peptides, providing insights into inter- and intracellular processes on the cell surface. The repertoire of presented peptides is continuously modulated by gene expression, transcription, translation, post-translational modification, antigen processing and presentation (Fortier et al., 2008; Caron et al., 2014; Bassani-Sternberg et al., 2015) and can be recognized by T cells. This enables the immune system to be constantly updated about inter- and intracellular disorders such as cancer transformation, virus or drug influences (Freudenmann et al., 2018; Nelde et al., 2018; Croft et al., 2019; Rajaraman et al., 2019).

Natural HLA-presented peptides have been isolated and sequenced using LC-MS/MS for almost three decades (Falk et al., 1991; Hunt et al., 1992; Ghosh et al., 2020). LC-MS/MS based peptide discovery enables to investigate the entirety of HLA-presented peptides. This method can be validated according to current pharmaceutical guidelines (Ghosh et al., 2020) and a high-throughput immunopeptidomics pipeline is feasible (Chong et al., 2018). Nowadays, this method is broadly applied and used in both personalized cancer vaccine development and biomarker discovery to support immunotherapy (Singh-Jasuja et al., 2004; Freudenmann et al., 2018; Calvo Tardón et al., 2019). Many cancer-, virus- and drug-induced HLA-presented peptides have been characterized, thus the suitability of this system for other forms of therapy seems obvious.

For centuries, therapies with implants of various materials have been developed (Saini et al., 2015). Nowadays, biomaterials should induce an immune response which does not initiate the removal of the foreign material, and they should not trigger excessive defensive reactions that might potentially damage the host tissue. They should not only imitate the function of the damaged tissue, but also initiate self-regeneration and gradually degrade after implantation. In addition to the increasing biomaterial design requirements of biocompatibility and bioactivity, regulatory requirements are also rising, especially for design and development as described in guidelines such as ISO 13485 and 21 CFR part 820. Therefore, it is of increasing importance for biomaterial development to understand the effects of biomaterials on the immune system in detail and to develop test systems that allow assessing and predicting of such effects.

Immune responses to implants can be induced by protein adsorption, complement activation, coagulation, neutrophil and macrophage adhesion, and activation by the exogenous material (Anderson, 1993). It is assumed that biomaterials function as adjuvants by inducing an immune response, recruiting and subsequently activating antigen-presenting cells (Matzelle and Babensee, 2004). This adjuvant-induced immune response is reminiscent of classical vaccination, in which adjuvants are used to generate immune responses against antigens, with HLA molecules playing a key role. This might indicate a relevant role for HLA molecules in the biomaterial implantation process. Many cases have been reported in which implanted materials are suspected to have triggered autoimmune diseases (Griem and Gleichmann, 1995; Brown et al., 1998; Rowley and Monestier, 2005; Alijotas-Reig, 2015; Suda et al., 2016; Colaris et al., 2017). Ultimately, this may be due to the adjuvant function of the material that has boosted T cells with specificity against HLA-presented self-peptides. Meanwhile, the opposite approach with biomaterials as adjuvants combined with antigens (Reddy et al., 2006; Jones, 2008) or attempts to cure autoimmune diseases with immunosuppressive biomaterials are being employed (Harrison, 2008; Prosperi et al., 2017).

The trend for the development of assays to estimate biomaterial-induced immune responses is moving from complex but insightful animal experiments to simple cell-based *in vitro* assays enabling high-throughput analysis. The high failure rates with frequent rejection of implants *in clinic* might be decreased using *in vitro* immune assays to predict the outcome prior to *in vivo* applications (Oliveira and Mano, 2014; Kohli et al., 2018; Lock et al., 2019). There are *in vitro* biomaterial immunogenicity assays applied based on immature human cell lines and peripheral blood mononuclear cells (PBMCs) to assess material-induced cell viability, maturation, activation, chemotaxis, and protein secretion (Smith et al., 2009; Anderson and McNally, 2011; Franz et al., 2011; Yahyouche et al., 2011; Kou et al., 2012; Spiller et al., 2014; Sussman et al., 2014; Lock et al., 2019; Przekora, 2019; Segan et al., 2020). An increased number of HLA molecules on the cell surface is an established inflammatory marker (Park and Babensee, 2012; Musson et al., 2015), suggesting the next step, to find out which peptides are presented to T cells in higher numbers after contact with the biomaterial.

In this study we present a novel *in vitro* assay that we developed to identify the first biomaterial-induced peptides in the immunopeptidome. Since monocytes are one of the initial immune cells to reach the implanted biomaterial through the bloodstream (Anderson et al., 2008; Ogle et al., 2016), we have investigated the influence of polyethylene, polyurethane, zinc, stainless steel, aluminum, copper and lipopolysaccharide (LPS) as biological control contact on THP-1 cells of after 24 h. We observed that material-monocyte contact leads to material-specific signatures in the immunopeptidome. There are multiple peptides originating from inflammation-, cellular stress-, autoantigen-, and fibrosis-associated proteins, which might be suitable biomarkers. Thus, the immunopeptidome has great potential to provide further insights into biomaterial-cell interaction, which can provide a deeper understanding of the biomaterial-induced immune response.

## Materials and Methods

### Materials

Since we aimed in this study to investigate whether different materials (polymers, metals) lead to modulations in the immunopeptidome, defined materials were selected according to ISO 10993-12, which recommends certain control materials for the biological evaluation of medical devices, including tests for implantation. For material incubation aluminum plates (aluminum), stainless steel 1.4301 plates (steel), zinc-plated washers (zinc washer), high-density polyethylene (RM-C, Hatano Research Institute/Food and Drug Safety Center, Japan), and polyurethane films containing 0.1% zinc diethyldithiocarbamate (RM-A, Hatano Research Institute/Food and Drug Safety Center, Japan) with a surface of about 1 cm^2^ with 1 mm thickness as well as copper and zinc sulfate solutions were used. We used four pieces of steel plates, zinc washers, RM-C and RM-A cuts and two, four or eight pieces of aluminum for the dose-response experiment. Except for the dose-response experiments, only the incubation with four aluminum plates was used in the evaluation. Copper and zinc sulfate solutions were used at 20 and 800 mg/l. Additionally, LPS from *Escherichia coli* 055:B5 (Sigma) was used as an inflammatory positive control at 50 ng/ml. Metal plates were cleaned by application of 2 min ultrasound in acetone, 2 min ultrasound in isopropanol and twice 2 min ultrasound in sterile water. All materials and solutions were sterilized by autoclaving. In the second and third assay, cells were incubated for 24 h with the materials and LPS as described in Supplementary Table 1.

### Cell Samples

The human acute monocytic leukemia cell line THP-1 (DSMZ ACC 16) was cultured in RPMI1640, containing 10% heat-inactivated fetal bovine serum (FBS) and 1% penicillin/streptomycin to a total number of 3 × 10^8^, 8 × 10^8^, and 8 × 4 × 10^8^ cells for assay I-III, respectively. For harvest, cells were centrifuged at 1,500 rpm for 15 min at 4°C, washed twice with cold PBS and aliquots containing 75 × 10^6^ cells were frozen and stored at −80°C until use. The cells were confirmed to be negative for mycoplasma *via* PCR.

### Immunoaffinity Purification of HLA-Presented Peptides

THP-1 cells were lysed in 10 mM CHAPS (Applichem)/PBS (Lonza) containing protease inhibitors (Complete, Roche). HLA class I and II-presented peptides were isolated using the pan-HLA class I-specific mAb W6/32 (Barnstable et al., 1978), the pan-HLA class II-specific mAb Tü-39, and the HLA-DR-specific mAb L243 (produced in-house) covalently linked to CNBr-activated Sepharose (GE Healthcare). HLA molecules and peptides were eluted by repeated addition of 0.2% trifluoroacetic acid (TFA, Merck). For peptide purification, ultrafiltration with centrifugal filter units (Amicon, Merck Millipore) was employed. Peptides were extracted and desalted using ZipTip C18 pipette tips (Merck), eluted in 35 μl 32.5% acetonitrile (AcN, Merck)/0.1% TFA, vacuum centrifuged to 5 μl, and resuspended in 25 μl of 1% AcN/0.05% TFA. Until LC-MS/MS analysis the peptide solutions were stored at −20°C.

### Analysis of HLA-Presented Peptides by LC-MS/MS

Immunopeptidome analysis was performed on a nanoflow high-performance liquid chromatography (nanoUHPLC, UltiMate 3000 RSLCnano, Dionex) on-line coupled to a LTQ Orbitrap XL tandem mass spectrometer (Thermo Fisher Scientific). For analysis, 5 μl peptide solution (20% of the eluted peptides per sample) were injected onto a 75 μm × 2 cm trapping column (Acclaim PepMap RSLC, Dionex) at 4 μl/min for 5.75 min. Peptide separation was performed at 50°C and a flow rate of 175 nl/min on a 50 μm × 25 cm separation column (Acclaim PepMap RSLC, Dionex) with a gradient ranging from 2.4 to 32.0% of AcN over the course of 90 min. Peptides were ionized by nanospray ionization and analyzed using a top five CID method with survey scans at 60,000 resolution and fragment ion detection in the ion trap operated at normal scan speed and limited mass range of 400–650 m/z (HLA class I) or 300–1500 m/z (HLA class II) with precursors of charge states 2+ and 3+ (HLA class I) or ≥2 (HLA class II) eligible for fragmentation. A total of five technical replicates were acquired per sample.

### Database Search and Spectral Annotation

The acquired LC-MS/MS data was processed against the human proteome included in the Swiss-Prot database^1^ (release September 27, 2013; containing 20,279 reviewed protein sequences) using the SEQUEST algorithm (Eng et al., 1994) embedded in the Proteome Discoverer (version 1.4, Thermo Fisher).

The precursor mass tolerance was set to 5 ppm and fragment ion mass tolerance was set to 0.5 Da with oxidized methionine allowed as the only dynamic modification with no restriction of enzymatic specificity. Percolator (Käll et al., 2007)-assisted false discovery rate (FDR) calculation was set at a target value of *q* ≤ 0.05 (5% FDR). HLA class I annotation was performed using the prediction algorithm NetMHCpan 4.0 as described in Jurtz et al. (2017); Ghosh et al. (2019) to determine the purity of peptide eluates in Supplementary Table 1. Venn diagrams were created using BioVenn (Hulsen et al., 2008), oxidized methionine was not considered. For label-free quantification of the relative HLA-presented peptides abundances all technical replicates were used for each sample. Relative quantification of peptides was performed by calculating the area under the curve of the corresponding precursor-extracted ion chromatograms in Proteome Discoverer (version 1.4, Thermo Fisher). The ratios of the mean areas of the individual peptides in the MS runs between conditions were calculated, and two-tailed *t*-tests implementing Benjamini–Hochberg correction were performed with R (v3.2).

### Cytokine Analysis Using a Luminex Multiplexed Bead-Based Sandwich Immunoassay

Following material incubation, the cytokine concentrations of the supernatants of THP-1 cells were analyzed. The change in cytokine concentrations after material and LPS incubation are given as fold change compared to the cytokine levels before material incubation, subtracting the fold changes of the untreated control. Levels of IL-1β, IL-1ra, IL-4, IL-8, IL-10, IL-12p70, GM-CSF, IFNγ, MCP-1, MIP-1β, TNF-α, and VEGF were determined using a set of “in-house developed” Luminex-based sandwich immunoassays each consisting of commercially available capture and detection antibodies and calibrator proteins. All assays were thoroughly validated ahead of the study with respect to accuracy, precision, robustness, specificity and sensitivity (European Medicines Agency, 2011; Food Drug Administration, 2018). Samples were diluted at least 1:4 or higher. After incubation of the pre-diluted samples or calibrator protein with the capture coated microspheres, beads were washed and incubated with biotinylated detection antibodies. Streptavidin-phycoerythrin was added after an additional washing step for visualization. For control purposes, calibrators and quality control samples were included on each microtiter plate. All measurements were performed on a Luminex FLEXMAP^®^ 3D analyzer system, using Luminex xPONENT^®^ 4.2 software (Luminex). For data analysis MasterPlex QT, version 5.0 was employed. To ensure proper assay performance, standard curve and quality control samples were evaluated according to internal criteria adapted to the Westgard Rules (Westgard et al., 1981).

## Results

### Immunopeptidomic Characterization of the Cellular Response to Materials

**FIGURE 1:**
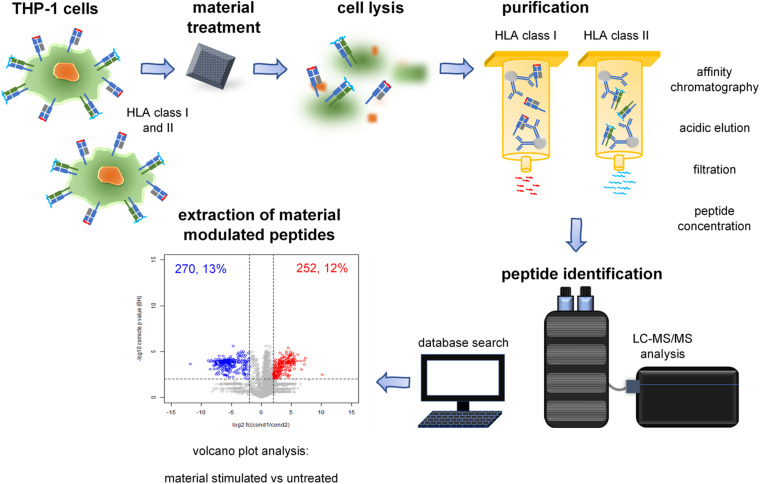
Immunopeptidomics of material incubated cells. Immunoprecipitation of HLA-peptide complexes, extraction of HLA class I- and II-presented peptides, and identification using LC-MS/MS. The individual steps are described in the illustrated pipeline.

Since the immunopeptidome has proven itself in biomarker discovery, we aimed at integrating this technology into biomaterial research (Figure 1). Currently there is intense research on new *in vitro* test systems for biomaterials. Therefore, our motivation was to develop an *in vitro* model based on the immunopeptidome that could provide a further dimension of information complementing currently available biomaterial research.

### THP-1 Cells Provide a Reliable Assay System for Immunopeptidomic Screening

In order to establish an *in vitro* monocyte test system, based on our experience, we selected the THP-1 leukemic monocyte cell line. THP-1 is a suspension cell line, which enables a simple large-scale cultivation. Almost every human individual has at least one of the HLA alleles of THP-1 (Supplementary Figure 1). The HLA class I allotypes comprise HLA-A^∗^02:01, A^∗^24:02, B^∗^15:11, B^∗^35:01, C^∗^03:03 and class II HLA-DPB1^∗^02:01, DPB1^∗^04:02, DQA1^∗^01:01, DQA1^∗^01:02, DQB1^∗^05:01, DQB1^∗^06:02, DRB1^∗^01:01, and DRB1^∗^15:01. These HLA enable a cumulative % of population coverage of 100% for one allele and 93% for two alleles. Immunopeptidome analysis of HLA class I- and class II-presented peptides resulted in high peptide yields for both classes (Supplementary Figure 2). Our standard immunopeptidomics pipeline analyzing five technical replicates per sample enabled the identification of almost all peptides identifiable by this method. In the fifth replicate we discovered less than 5% novel unique peptides for HLA class I and less than 10% for class II in comparison with the previous four technical replicates.

### Separate Cultivation of Cells Originating From the Same Batch Results in Distinct Differences in the Immunopeptidome

To develop a reliable test system, we first examined the boundaries of immunopeptidome analysis. Since the immunopeptidome is very sensitive, we first wanted to investigate the influence of different culture conditions on the immunopeptidome of THP-1 cells. Starting from a frozen batch in two experiments (assay I and II), we cultured three THP-1 populations up to a cell count of 1 × 10^8^ cells. In the first experiment, the cells were cultured independently in flasks (assay I) until harvest and LC-MS/MS analysis. In the second experiment, the cells were cultured together in one population (assay II) and were subsequently cultured independently for 2 days until harvest and LC-MS/MS analysis (Supplementary Figure 3).

Interestingly, the number of HLA class I- and II-presented peptides in the three batches of assay I (batch 1–3) did also significantly differ from assay II (batch 4–6), although the cells in assay I and II were derived from the same frozen cell batch (Supplementary Figure 2). Regarding the relative standard deviation (%RSD) of the presented peptides of batch 1–3 and 4–6, the %RSD of batch 4–6 is only half as high for HLA class I and equal for class II. For HLA class II, the %RSD of batch 4–6 is as high as for batch 1–3, although only half as many peptides are presented.

Overlap analysis of the individual batches 1–3 revealed large differences in the peptides presented for HLA class I and II compared to the batches 4–6 (Supplementary Figures 4, 5A,B). An overlap of the batches 1–3 combined with the three batches 4–6 combined led to an even lower proportion of shared peptides of 37% HLA class I and 15% class II peptides (Supplementary Figures 6A,B). This combined overlap is low regarding the total number of peptides. However, in relation to the peptides of the three batches with the lowest peptide number the percentage of shared peptides was comparable to the percentage in the independently cultivated batches 1–3.

### Materials for Cytotoxicity Induction Result in Distinct Alterations of the Immunopeptidome After One-Day-Stimulation

In order to investigate whether materials induce general differences in the immunopeptidome compared to untreated cells, we incubated cells with reliable reference materials for cytotoxicity in a 24 h-incubation setup (assay II, Supplementary Figure 3). In accordance to ISO 10993-12, the recommended positive/negative control materials for cytotoxicity and implantation studies were applied. The positive control, RM-A, which induces moderate cytotoxicity and the negative control, RM-C, were used. The HLA class I and II peptide yields and %RSD of identified peptides of the technical and biological replicates of the RM-A and RM-C treated THP-1 cells were comparable with that of untreated cells (Supplementary Figures 7A,B and Supplementary Table 2: identified peptides). A combination of the material-incubated samples with the untreated samples also revealed no differences in the %RSD of the identified peptides compared to the untreated samples alone. As previously demonstrated for untreated samples, five replicates were sufficient to identify most peptides also after material incubation.

To further investigate a global material-induced effect in the presented HLA class I or II peptides, each material-incubated sample was overlapped with the untreated samples and compared to the overlap of untreated samples with each other (Supplementary Figure 8A). Almost no significant material-induced alteration was visible in the global analysis of all shared and unique peptides, except for shared HLA class II peptides of one RM-A-incubated sample.

Since materials did not lead to a global material-induced effect in the immunopeptidome, we used comparative profiling to identify possible material-exclusive peptides and volcano plot analysis for semi-quantitative analysis to identify up- or down-modulated peptides.

By subtracting the peptides of the untreated samples, we determined the treatment-exclusive peptides of the material incubated samples. We investigated whether these exclusive peptides do overlap more after incubation with two similar materials compared to different materials. Overlap of material-exclusive peptides between RM-A or RM-C treatments did not reveal a higher proportion of shared peptides compared to the overlap of RM-A- and RM-C-treated samples (Supplementary Figure 8B). Since contact with materials is likely to induce substantially fewer altered intracellular processes compared to cancer transformation or virus infection, comparative profiling did not lead to the identification of new exclusive peptides.

**FIGURE 2:**
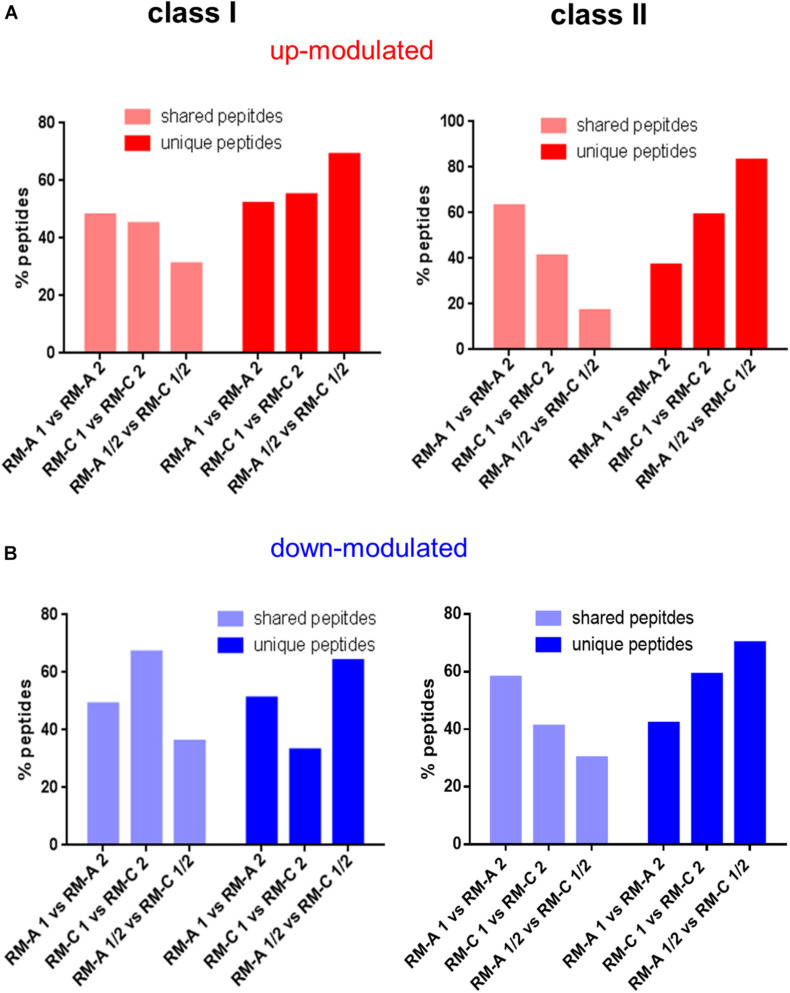
Screening for peptides significantly altered by materials. Shared and unique peptides after overlapping of the peptides that are significantly up- (**A**; red) or down-modulated (**B**; blue) after RM-A or RM-C incubation compared to the three untreated samples (batch 4–6) for HLA class I and II. The significantly up- and down-modulated peptides after material incubation (material-associated peptides) were determined using the semi-quantitative volcano analysis of the modulation in the relative abundances of HLA-presented peptides on THP-1 cells comparing material and LPS treated cells with untreated cells (≥log_2_ 2-fold-change in abundance with *p* < 0.01).

We next searched for significantly up- or down-modulated HLA-presented peptides performing semi-quantitative volcano plot analysis. In the analysis, significantly up- or down-modulated HLA-presented peptides by RM-A or RM-C incubation were overlapping more between two samples both incubated with RM-A or RM-C than a RM-A and RM-C incubated sample, for HLA class I and II (Figures 2A,B and Supplementary Figure 9: visualized volcano plots and Supplementary modulated peptides). RM-A and RM-C incubation seemed to modulate peptides in the immunopeptidome, which are specific for the respective material. These modulated peptides appearing after material incubation will be referred to as material-associated peptides.

### Material-Associated Peptides Originate From Source Proteins With Manifold Functions

In addition to RM-A and RM-C, THP-1 cells were treated with aluminum plates (aluminum), stainless steel plates (steel), galvanized steel plates (zinc washer), dissolved zinc sulfate (zinc sulfate) and dissolved copper (copper) to investigate the manifold influences of different materials. LPS was used as biological positive control.

Performing semi-quantitative volcano plot analysis of incubated samples compared to untreated samples, a closer look at the source proteins of the top five significantly up- or down-modulated peptides revealed several shared protein features (Supplementary Tables 3A,B, S2: identified and modulated peptides). Among the multifarious top five proteins, possible roles associated with differentiation, wound healing, cytoskeleton or cell adhesion, cell migration, phagocytosis, metals, apoptosis or autophagy, immune response or inflammation, and stress response could be assigned by UniProt Keywords and Gene Ontology terms (UniProt, October 2019), which might be associated with cellular responses to biomaterial contact. For HLA class I it was particularly interesting that many proteins are associated with metal binding, although only 8% of the human proteome are associated with that function (UniProt, October 2019). However, the metal-associated proteins are not only present in the up- or down-modulated top five of metal incubated samples, but also in metal free RM-C and LPS-incubated samples. Besides metal binding, ribosomal proteins were enriched for HLA class II (Supplementary Table 3B), which usually make up only 3% of proteins in the human proteome (UniProt, October 2019).

Regarding the modulated peptides, all tested materials had an effect on the immunopeptidome comparable to the number of peptides significantly modulated by LPS (Supplementary Figure 9). In analyses of the cytokine content of the supernatants of the incubated cells (Supplementary Table 4), significant increases of IL-4, IL-8, GM-CSF, IFN-y, MCP-1, MIP-1β, TNF-α, and IL-1β concentrations were measured after LPS incubation, while materials only revealed reduced VEGF levels after zinc sulfate incubation and increased IL-8 concentrations after copper incubation. The detected changes in cytokine concentrations in the supernatant after material treatment are consistent with the literature (Kassoff et al., 2001; Bar-Or et al., 2003; Li et al., 2014) and LPS contamination of the materials can be excluded.

### Material-Associated Peptides Differ According to Material Stimulation

An overlap of the significantly up- or down-modulated peptides after incubation with RM-A, RM-C, and zinc washer incubation in assay II and aluminum, copper, LPS, steel, and zinc sulfate in assay III led to distinct overlaps between the conditions for HLA class I and II (Supplementary Tables 5A,B). We must point out that due to the cultivation effects on the immunopeptidome (demonstrated in the previous “Results” section), the results of the assays II and III cannot be directly compared with each other. In the case of significantly up-modulated HLA class I-presented peptides (Supplementary Table 5A) mainly aluminum and LPS in assay III and RM-A and RM-C in assay II did overlap *vice versa*. The copper-induced peptides unusually overlapped evenly with the other conditions in assay III. Interestingly, the induced peptides of metal treated samples had a high percentage of shared peptides with LPS, which in the case of zinc sulfate even exceeded other metals. Regarding the significantly down-modulated HLA class I-presented peptides in assay III, aluminum and steel mostly overlapped *vice versa* and the percentage of shared peptide with LPS was lower compared to the other metals. In assay II, there was a high overlap of zinc washer and the zinc containing RM-A-induced peptides.

Regarding HLA class II, the highest percentage of shared peptides was between the significantly up-modulated peptides of aluminum and steel (Supplementary Table 5B). Again, the metal-associated peptides overlapped strongly with those induced by LPS. Compared to HLA class I, the overlap with aluminum in assay III was lower here, whereas the aluminum-associated peptides overlapped strongly with other conditions. In assay II mainly RM-A- and zinc washer-induced peptides overlapped *vice versa*. In the case of significantly down-modulated peptides, the overlap of aluminum and zinc sulfate-induced peptides was the highest, but not the other way around. It is striking that induced peptides of all conditions in assay III overlapped particularly with zinc sulfate. In assay II mainly RM-A- and zinc washer-induced peptides overlapped *vice versa*, but also RM-A and RM-C.

### Material- and LPS-Associated Peptides Originate From Source Proteins Related to Fibrosis, Autoimmune Diseases, Cytotoxicity, Inflammation, and Cellular Stress

**FIGURE 3:**
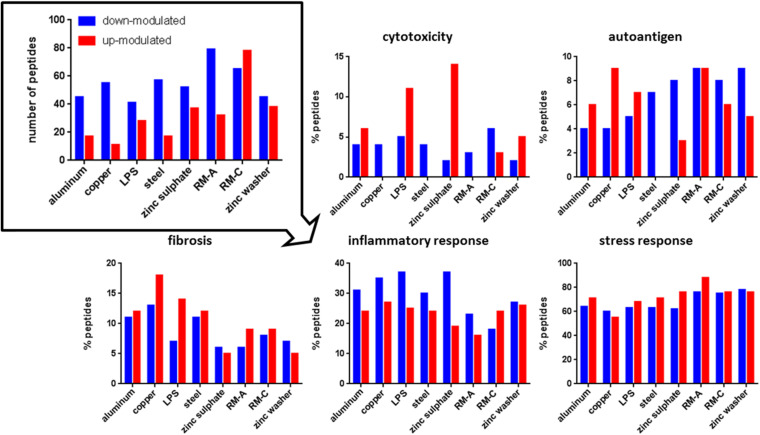
Significantly modulated peptides after material and LPS incubation. Total number of significantly up- (red) or down-modulated (blue) HLA class I peptides after material and LPS incubation (top left). Percentage of these peptides derived from source proteins associated with cytotoxicity, autoantigen, fibrosis, inflammatory, and stress response in UniProt. RM-A and RM-C duplicates were combined. The significantly up- and down-modulated peptides after material incubation (material-associated peptides) were determined using the semi-quantitative volcano analysis of the modulation in the relative abundances of HLA-presented peptides on THP-1 cells comparing material and LPS treated cells with untreated cells (≥log_2_ 2-fold-change in abundance with *p* < 0.01).

**FIGURE 4:**
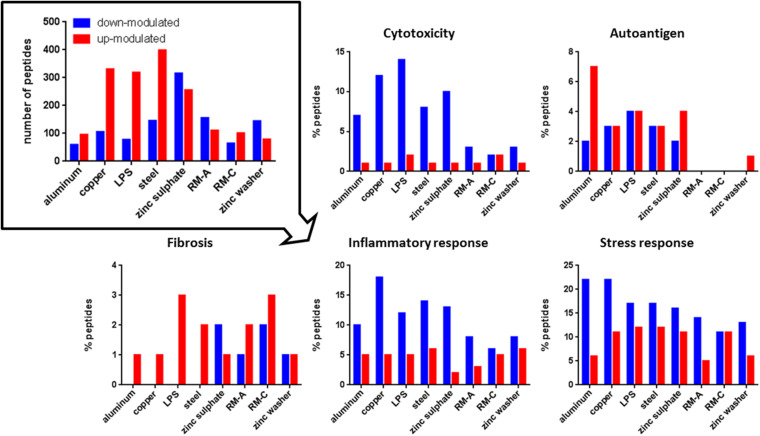
Significantly modulated peptides after material and LPS incubation. Total number of significantly up- (red) or down-modulated (blue) HLA class II peptides after material and LPS incubation (top left). Percentage of these peptides derived from source proteins associated with cytotoxicity, autoantigen, fibrosis, inflammation, and stress response in UniProt. RM-A and RM-C duplicates were combined. The significantly up- and down-modulated peptides after material incubation (material-associated peptides) were determined using the semi-quantitative volcano analysis of the modulation in the relative abundances of HLA-presented peptides on THP-1 cells comparing material and LPS treated cells with untreated cells (≥log_2_ 2-fold-change in abundance with *p* < 0.01).

The source proteins of the individual presented peptides were analyzed to gain insights into the biological meaning of the altered peptide abundances in the immunopeptidome after material incubation. We were particularly interested in proteins associated with cytotoxicity, autoimmune diseases, fibrosis, inflammation, and cellular stress in order to assess biomaterial influences more precisely (protein annotations obtained from UniProt, October 2019). Figures 3, 4 illustrate the number of significantly up- or down-modulated peptides after incubation derived from source proteins with the previously mentioned associations. In each incubation setting, peptides of these source proteins were identified. For HLA class II, significantly more up- or down-modulated peptides were detected after each incubation compared to HLA class I. However, more HLA class I peptides appear to be derived from proteins with associations of interest. All significantly modulated peptide numbers after material incubations were comparable with LPS incubation for all protein associations of interest.

Regarding cytotoxicity-associated HLA class I peptides, a high number of significantly up-modulated peptides after LPS and zinc sulfate incubation were identified. Concerning the known cytotoxic controls RM-A and RM-C, the less cytotoxic control RM-C led to more significantly modulated peptides in our assay. In the case of HLA class II, the significantly down-modulated peptides outweighed the significantly up-modulated peptides and here the most down-modulated peptides were identified after LPS, copper and zinc sulfate incubation. With respect to autoantigen-associated peptides, no up-modulated peptides were found after steel incubation for HLA class I and almost no significantly modulated peptides after RM-A, RM-C, and zinc washer treatment for HLA class II. Especially in case of aluminum there was a high number of up-modulated peptides for HLA class II. Regarding the fibrosis-associated peptides, copper led to most significant modulations for HLA class I and for HLA class II, only a few significantly modulated peptides were detected. In the case of inflammatory response and stress response, many significantly modulated HLA class I and II peptides were found after each incubation. In HLA class II, regarding cytotoxicity, inflammatory and stress response, the significantly down-modulated peptides seemed to predominate.

The associated source proteins that occurred after most incubations with different materials are depicted in Supplementary Tables 6, 7. Interestingly, regarding the HLA class I peptides after the incubations of assay III, one found many source proteins that occurred after multiple incubations, both with LPS and metals. The protein Bax inhibitor 1, TMBIM6, appeared in assay II after each incubation in the form of different peptides. In the case of HLA class II, for the top source proteins, which occurred after most incubations, many different peptides were detected. The source protein amyloid-beta precursor protein, APP, appeared after each incubation in the form of various peptides.

### Metal-Associated Peptides Do Not Appear to React in a Dose-Dependent Way

In order to investigate whether increased biomaterial contact led to increased changes in the immunopeptidome or whether the material-associated peptides were modulated, THP-1 cells from assay III were incubated with two, four, and eight aluminum plates. There was no general increase in the number of material-associated peptides and only the significantly down-modulated peptides in HLA class I and the significantly up-modulated peptides in class II had increased in number (Supplementary Table 8). The relative overlap of aluminum-induced peptides in the three aluminum incubated samples was high for HLA class I (up-modulated: 24%, down-modulated: 32%), but low for class II (up-modulated: 7%, down-modulated: 10%).

In terms of quantity, assuming a slope of at least ±0.5 fold change per condition from two to eight aluminum plates, there were only four altered peptides among the significantly down-modulated peptides for HLA class I (Supplementary Table 9) and for HLA class II, three altered peptides among the significantly up-modulated peptides and two altered peptides among the significantly down-modulated peptides. Compared to the number of aluminum-induced peptides shown in Supplementary Table 8, the number of up- or down-modulated peptides was negligibly small and there is probably no dose-dependent effect on the aluminum-associated peptides in biomaterial treatment.

### Comparison With Known Inflammatory Controls Such as LPS Allows an Evaluation of the Materials With Regard to Inflammation

As the immune response to biomaterials is very diverse and not yet fully understood (Mariani et al., 2019), it is difficult to make assumptions about the influence of a particular biomaterial on the immune system. For biological controls such as LPS, there are many consistent results that clearly indicate LPS as endotoxin that leads to inflammatory and cellular stress response and is cytotoxic (Schumann, 1992; Triantafilou and Triantafilou, 2005). Therefore, we have overlapped the peptides of cytotoxicity-, inflammatory- and cellular stress response-associated source proteins that emerged after incubation with the materials from assay III, with the LPS-induced peptides (Table 1). The HLA class I and II presented peptides were affected differently. For HLA class I, aluminum incubation was closest to LPS, whereas for HLA class II, aluminum was the most diverse. The induced changes after steel incubation were quite similar to LPS in both HLA class I and II. Since the immunopeptidome provides a versatile view on presented source proteins from various pathways, suitable controls such as LPS or IFN-γ (Chong et al., 2018) can be used to characterize and rank the materials more precisely.

**TABLE 1:** Similarity of top modulated HLA class I and II peptides with LPS.

Incubation	Cytotoxicity	Inflammatory response	Stress response
**Class I**			
LPS	100%	100%	100%
Aluminum	60%	68%	49%
Steel	40%	68%	49%
Copper	40%	59%	42%
Zinc sulfate	20%	59%	44%
**Class II**			
LPS	100%	100%	100%
Steel	71%	81%	49%
Copper	65%	62%	55%
Zinc sulfate	47%	38%	29%
Aluminum	12%	23%	16%

*Proportion of cytotoxicity-, inflammatory, and stress response-associated material-modulated peptides among LPS modulated peptides in assay III.*

## Discussion

In order to obtain a holistic view of the interactions between a biomaterial and the human body, a diverse suite of assays should be performed. Compared to the commonly employed assays, the examination of the immunopeptidome offers entirely new insights into the nature of biomaterials on biological systems. It reveals the signals that are passed on by antigen presenting cells (APCs) to T cells, thus shaping the adaptive immune response to biomaterials. Although research in the field of immunopeptidomics has been going on for decades, little is known about the biological functions of the thousands of peptides presented on the cell surface. Virus-, cancer-, and autoantigen-associated peptides have been the most intensively studied categories to date (Falk et al., 1991; Hunt et al., 1992; Lemmel et al., 2004; Wahlström et al., 2009; Bassani-Sternberg et al., 2016; Ternette et al., 2016; Zhou et al., 2017; Marcu et al., 2019; Rajaraman et al., 2019). Here, we have started with the basic questions whether materials have an influence on the immunopeptidome. Are there material-associated changes in the peptide composition and are they related to inflammation, fibrosis and cellular stress?

The goal of this study was to investigate the first reaction by the immunopeptidome after biomaterial contact. Monocytes are one of the first cells reaching the implanted material, thus a THP-1 cell model was chosen. THP-1 cells present a high number of both HLA class I and II peptides. Due to the dynamics and versatility of the immunopeptidome, materials and comparative controls under investigation should be used for incubation of cells from the same cultured cell population, to minimize individual cultivation effects. Currently, the best comparability can be achieved by reproducible high-throughput methods in 96-well format and a validated LC-MS/MS pipeline (Chong et al., 2018; Ghosh et al., 2020).

Upon incubation with materials, we would expect only weak changes in the immunopeptidome compared to the considerable influence of viral infections and cancer transformations, due to new signal cascades and newly synthesized proteins. In order to detect minor influences of materials on the immunopeptidome, first the cytotoxic influence of materials was examined, one of the most decisive points whether a material can be implanted (Rebelo et al., 2017). After 24 h of incubation with cytotoxic RM-A and non-cytotoxic RM-C, there were significantly up- and down-modulated material-associated peptides, that differed between the cytotoxic and non-cytotoxic material. Apart from these material-modulated peptides, the total number of peptides presented was not affected. The number of novel appearing or dwindling peptides after material incubation corresponded to natural fluctuations. Cytotoxicity is usually analyzed *via* morphological assessment, cell viability and proliferation assays, and functional assays such as determination of inflammatory markers, glutathione and heat shock proteins, as well as apoptosis assays (Omidi et al., 2017). The discovery of these significantly up- and down-modulated cytotoxicity-associated peptides might add an additional tool to the current set of methods used to assess cytotoxicity. For example, when analyzing biopsy tissue, the current sets of methods are difficult to employ for cytotoxicity assessment. Examining HLA-presented peptides related to cytotoxicity could provide advantages when dealing with tissues that have been processed or fixed following biopsy.

Besides RM-A and RM-C, material-associated peptides were identified for each tested material. These peptides and their source proteins were identified to be related to the type of biomaterial that the cell was in contact with. The source proteins of these material-associated peptides differed depending on the material treatment. In the case of HLA class I, the peptides of metal-associated proteins appeared to be enriched in the top metal modulated peptides, but also in the non-metallic materials. In HLA class II, the number of peptides from ribosomal proteins in the top modulated peptides were particularly striking. Since little is known about the intracellular changes after biomaterial contact, it is difficult to make a more precise interpretation. In order to survive, cells have to adapt to the new environment created by the added materials. Most of the top modulated peptides are derived from metal-associated proteins, which have ion transporter functions that play an important role in the regulation of metal ion levels (Bird, 2015). These proteins might be elevated by the new surroundings. The increased occurrence of ribosomes after biomaterial contact may indicate an adaptation of gene expression and translation regulation to the new environment (Liu and Qian, 2014). In immunopeptidomics, the significant quantitative changes in peptide presentation can only provide information about the peptides presented. Assumptions about proteins cannot be made, since, for example, increased protein synthesis or increased protein degradation can lead to an increased presentation of peptides (Bassani-Sternberg et al., 2015). Ultimately, the materials lead to specific changes in peptide presentation, indicating that the immune system might react in a way that could potentially lead to a host-induced response of the adaptive immune system, e.g., autoimmunity. These immunopeptidomic alterations could contribute to local inflammation of a biomaterial and thus to implant failure (Shakya and Nandakumar, 2017; Mariani et al., 2019). Autoreactive T cells against HLA-presented self-antigens are key players in autoimmune diseases and act as regulatory and effector cells (Khan and Ghazanfar, 2018). Therefore, it is important to study the material-associated peptides presented after material incubation. Especially in case of immunocompromised patients with an implant where autoreactive T cell effects may not be attenuated (Khan and Ghazanfar, 2018).

These material-associated peptides did not seem to react in a dose-dependent manner to material incubation. An incubation with increasing amounts of aluminum plates indicated that aluminum-associated peptides were presented and overlapping in the incubated samples, but they did not seem to react to the increased aluminum incubation. Due to the increased addition of material, more contact between the THP-1 cells and the materials is likely to have resulted, along with an increase in release of any ions that may have been released from the material. However, it should be mentioned that passivation of the aluminum may have occurred, or saturation of aluminum ions in the media may have limited any potential dose-dependent effect.

The HLA class I and II presented material- and LPS-modulated peptides were differently affected after incubation. A striking feature of the down-modulated peptides in HLA class II was the high overlap of all assay III incubated peptides with zinc sulfate. Most of the aluminum-, copper-, steel- and LPS-associated HLA class II peptides also appeared to be induced by zinc. An anti-inflammatory and immunomodulating effect of zinc is known and in the case of HLA class II intracellular zinc might have an influence on the transport of the HLA class II molecules to the plasma membrane (Bonaventura et al., 2015; Wong et al., 2015; Maares and Haase, 2016).

A broad look at the source proteins of the material-associated peptides revealed that many proteins are related to fibrosis, autoimmune diseases, cytotoxicity, inflammation, and cellular stress, both for HLA class I and II. The number of peptides derived from these source proteins alone did not indicate any obvious material-specific differences. The treatments led predominantly to quite similar peptide numbers in the respective groups. Compared to the tested materials, LPS-induced peptides did not seem to differ in cytotoxicity, inflammation, and cellular stress. The cytotoxic positive control RM-A did also not exceed RM-C in the number of peptides from cytotoxicity associated proteins. Differences were found in the individual peptides. Most of the source proteins of the material-associated peptides were shared between a few incubations with different materials and only the peptides originating from the cellular stress-associated proteins TMBIM6 and APP were significantly down-modulated after each incubation. Both proteins have multiple functions and are probably engaged in many cell stress-inducing processes. TMBIM proteins are involved in organelle-specific cell death mechanisms, pathogen-host reactions, pH-dependent regulation of Ca^2+^ signal and reshaping of the signal in cancer (Liu, 2017). APP is known to interact with many metals such as zinc and copper. It is involved in copper homeostasis and oxidative stress through copper ion reduction (Baumkötter et al., 2014; Istrate et al., 2016). Furthermore, it is involved in cellular toxicity and inflammatory processes have an important role in APP expression (Brugg et al., 1995; Park et al., 2009).

It is noticeable that many material-associated peptides were shared with the inflammatory control LPS, which is cytotoxic, induces an inflammatory and cellular stress response and could therefore use many signaling pathways that are also induced by material incubation (Franz et al., 2011). Endotoxins such as LPS, might be a suitable control to identify undesirable effects of materials. An overlap of the material-associated peptides from the cytotoxicity, inflammation, and cellular stress associated source proteins with the peptides modulated by LPS allows to estimate which materials might act in a manner comparable to LPS. Many of these LPS-modulated peptides were also modulated by aluminum and steel for HLA class I and steel and copper for HLA class II. On the contrary zinc sulfate modulated a relatively small number of these peptides. Toxic effects have been observed for all tested materials, but so far only the metal hypersensitivity of nickel, cobalt and palladium is suspected to be caused by TLR 4, which also recognizes bacterial LPS (Schrand et al., 2010; McKee and Fontenot, 2016; US FDA, 2019). Based on the HLA class I and II presented peptides, stainless steel seems to have the most resembled LPS-like effect.

## Conclusion

Our first attempt to integrate immunopeptidomics into material testing revealed that materials modulate the immunopeptidome and material-associated peptides are presented. The peptides occur after contact with both metal and polymer. This is consistent with the theory that no material can enter the body unobserved and that all non-body biomaterials will eventually induce an immune reaction (Franz et al., 2011).

Here, we present one strategy to identify material-associated peptides. Immunopeptidome analysis revealed a large number of material-associated peptides, which are presented to immune cells and possibly modulate the adaptive immune response. The difficulty remains to estimate the functional role of the individual peptides in order to judge whether materials will be rejected or not. Is the peptide presented due to inflammatory or fibrotic reasons? By means of extensive comparisons with thoroughly investigated controls, such as LPS, comparisons with controls having different mechanisms of action will allow more precise characterization of material effects in the future. As a follow-up to the control materials used in this study, which have a significant impact on the body, other materials currently used for implantation could be assessed in future for their impact on the immunopeptidome.

Since the LC-MS/MS pipeline can now be validated, new mass spectrometers allow an even higher sensitivity, and the 96 well format has proven to be feasible (Chong et al., 2018; Ghosh et al., 2020), the immunopeptidomic pipeline can in future be performed with an increased robustness, recovery rate, sample throughput, and comparability. This will enable to precisely determine the frequently occurring material-associated peptides and enable to identify the roles of these peptides through extensive comparisons with established controls. Subsequently, specific peptides could be determined to possibly enable a prediction of the acceptance or rejection of implants based on the *in vitro* analysis of the immunopeptidome.

## Data Availability Statement

The mass spectrometry data have been deposited to the ProteomeXchange Consortium (http://proteomecentral.proteomexchange.org) *via* the PRIDE (Vizcaíno et al., 2014) partner repository with the dataset identifier PXD019258.

## Author Contributions

MG, SSt, TJ, SSe, H-GR, CS, and HH designed the research. MG, LMä, MJ, LF, and LMü performed the research. MG and LB analyzed the data. MG wrote the manuscript. All authors revised the manuscript.

## Conflict of Interest

The authors declare that the research was conducted in the absence of any commercial or financial relationships that could be construed as a potential conflict of interest.

## Footnotes

1 http://www.uniprot.org
