# Supplementary material for: The Impact of Biomaterial Cell Contact on the Immunopeptidome
Source: Front Bioeng Biotechnol. 2020 Dec 16;8:571294. doi: 10.3389/fbioe.2020.571294 (PMC7773052; doi:10.3389/fbioe.2020.571294)
Supplement: Supplementary file 1 [file Data_Sheet_1.zip › Supplemental Figure S9.PDF]

Supplemental Figure S9

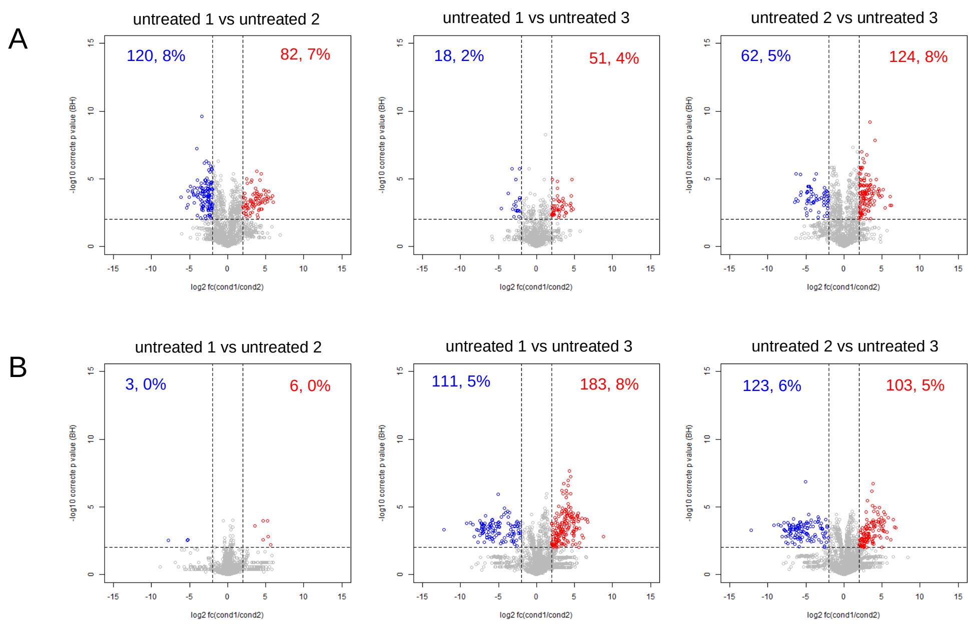

Volcanos part 1: HLA class I presented peptides in untreated THP-1 cells of assay I (A, batch 1-3) and assay II (B, batch 4-6).

Supplemental Figure S9

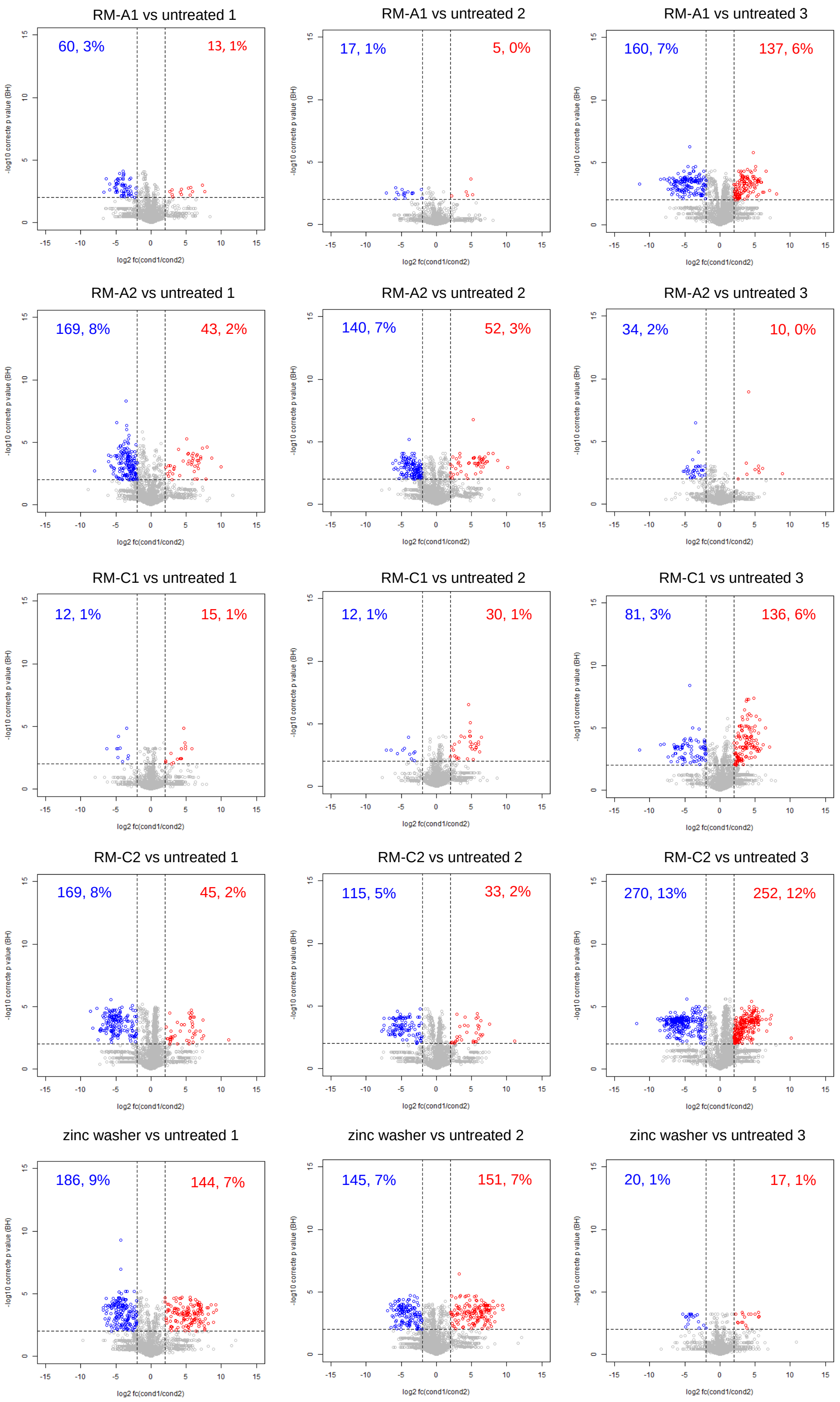

Volcanos part 2: HLA class I presented peptides in untreated (batch 4-6), RM-A- and RM-C-stimulated THP-1 cells in assay II.

Supplemental Figure S9

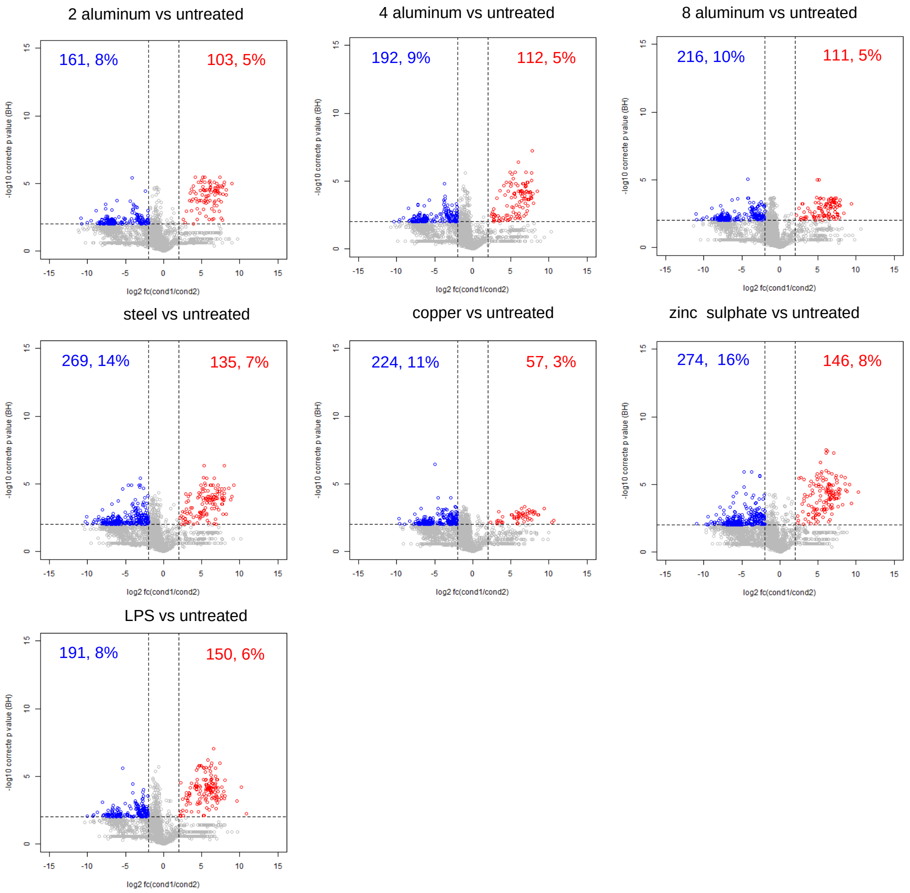

Volcanos part 3: HLA class I presented peptides in untreated, aluminum-, steel-, copper-, zinc sulphate- and LPS-stimulated THP-1 cells in assay III.

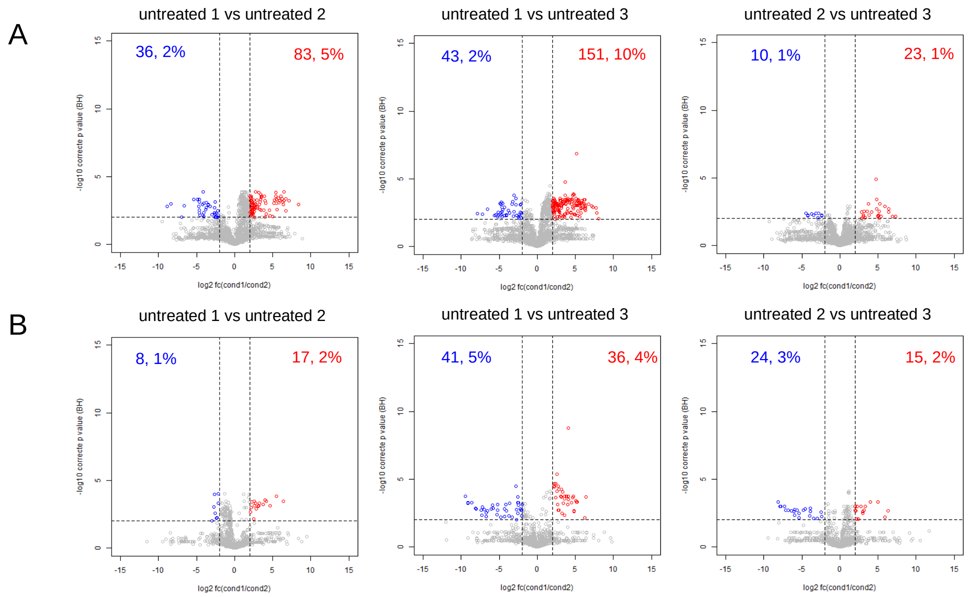

Volcano 4: HLA class II presented peptides in untreated THP-1 cells of assay I (A, batch 1-3) and assay II (B, batch 4-6).

Supplemental Figure S9

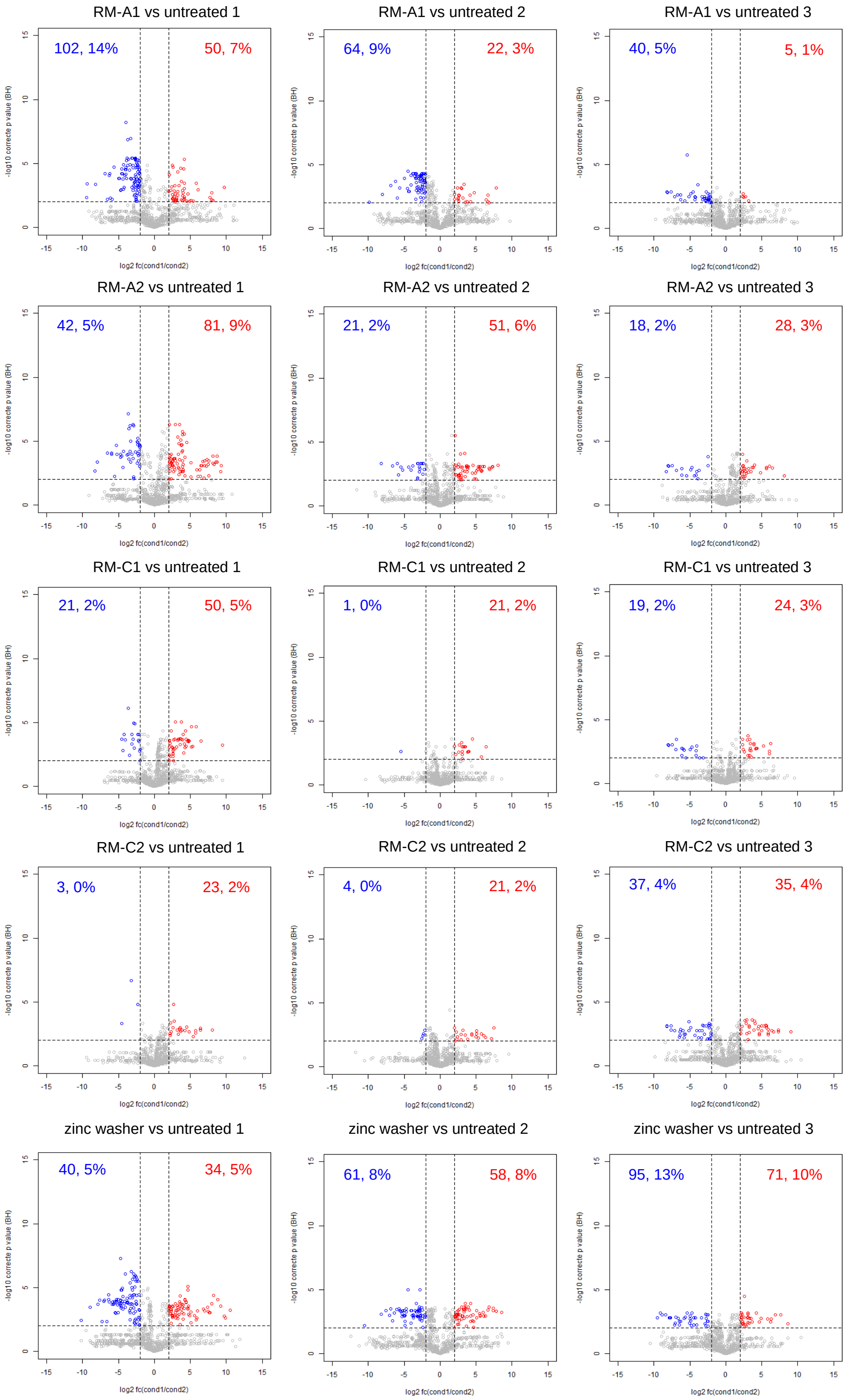

Volcanos part 5: HLA class II presented peptides in untreated (batch 4-6), RM-A- and RM-C-stimulated THP-1 cells in assay II.

Supplemental Figure S9

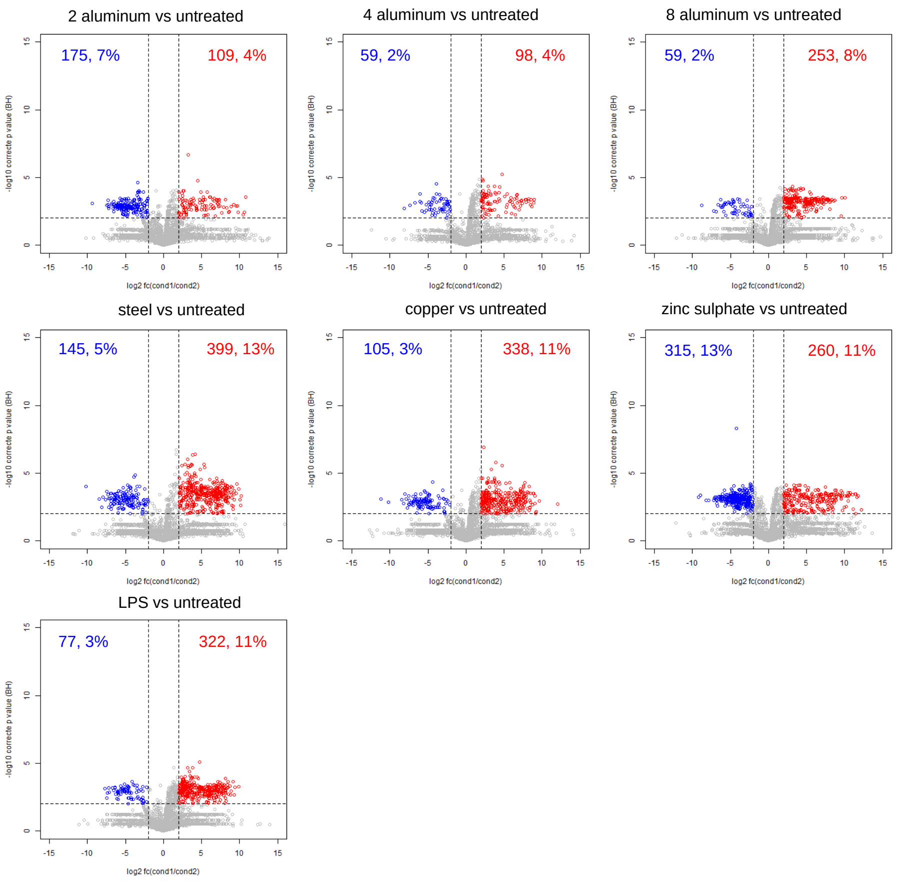

Volcanos part 6: HLA class II presented peptides in untreated, aluminum-, steel-, copper-, zinc sulphate- and LPS-stimulated THP-1 cells in assay III.
