# Supplementary material for: The Impact of Biomaterial Cell Contact on the Immunopeptidome
Source: Front Bioeng Biotechnol. 2020 Dec 16;8:571294. doi: 10.3389/fbioe.2020.571294 (PMC7773052; doi:10.3389/fbioe.2020.571294)
Supplement: Supplementary file 1 [file Data_Sheet_1.zip › Supplemental Table Captions and Figures.PDF]

Supplementary Material

1 Supplemental Tables and Figures

1.1 Supplemental Tables Legends

Supplemental Table S1: Sample summary. Overview of experiments, incubations, number of cells before material incubation, acquired LC-MS/MS measurements, analyzed HLA classes, identified unique peptides, purity (percentage of peptides matching the peptide motif of the respective HLA allotype) and raw file name in the online-repository.

Supplemental Table S2: HLA class I- and II-presented peptides and material-associated peptides. Total identified HLA class I and II peptides of the samples in assay II and III and all material-associated peptides, which are significantly up- or down-modulated after material incubation.

Supplemental Table S3: Top material-associated HLA class I and II peptides and corresponding source proteins. Top five significantly up- and down-modulated peptides and their source proteins after each material and LPS incubation. The peptide sequence, source protein, exclusivity of the peptide for this material incubation, the fold change after incubation compared to untreated, the molecular function, biological process, and ligand description of the UniProt Keywords and material associated functions from the nine categories we have selected (derived from the Gene Ontology terms: differentiation (D), wound healing (WH), cytoskeleton or cell adhesion (C), cell migration (CM), phagocytosis (P), metals (M), apoptosis or autophagy (A), immune response or inflammation (IR), and stress response (SR)) are given. RM-A and RM-C duplicates were combined.

Supplemental Table S4: Influence of material incubation on cytokine secretion. After material incubation, cytokine concentrations of THP-1 cell supernatants were analyzed. The change in cytokine concentrations after material and LPS incubation are given as fold change compared to the same cells before material incubation, subtracting the fold changes of the untreated control. RM-A and RM-C duplicates were combined. Fold change difference to untreated: **-** (-2 fold), **+** (2 fold), **++** (5-10 fold), **+++** (10-50 fold), and **++++** (>50 fold).

Supplemental Table S5: Overlap of modulated material- and LPS-associated HLA peptides. Overlap of the significantly up-modulated (top) and down-modulated (bottom) HLA class I (A) and II (B) peptides by the material incubations in the second column with the different material incubations in the top row. RM-A and RM-C duplicates were combined.

Supplemental Table S6: Top modulated HLA class I peptides and source proteins from the associations of interest. The top significantly up- and down-modulated peptides from the fibrosis-, autoantigen-, cytotoxicity-, inflammatory- and stress response-associated source proteins found most frequently in the aluminum-, copper-, LPS-, steel-, zinc sulphate-, RM-A-, RM-C-, and zinc washer-incubated samples. RM-A and RM-C duplicates were combined.

Supplemental Table S7: Top modulated HLA class II peptides and source proteins from the associations of interest. The top significantly up- and down-modulated peptides from the fibrosis-, autoantigen-, cytotoxicity-, inflammatory- and stress response-associated source proteins found most

frequently in the aluminum-, copper-, LPS-, steel-, zinc sulphate-, RM-A-, RM-C-, and zinc washer-incubated samples. RM-A and RM-C duplicates were combined.

Supplemental Table S8: Influence of different aluminum concentrations on aluminum-associated peptides. Number of aluminum-associated HLA class I and II peptides with significantly modulated HLA-presentation after incubation with two, four or eight aluminum platelets and modulated in at least two samples.

Supplemental Table S9: Influence of different aluminum concentrations on aluminum-associated peptide abundances. Aluminum-associated HLA class I and II peptides up- or down-modulated with a fold change of 0.5 from two to eight aluminum platelets. N/A: no indication possible because the source protein of the peptide is not specific.

1.2 Supplemental Figures

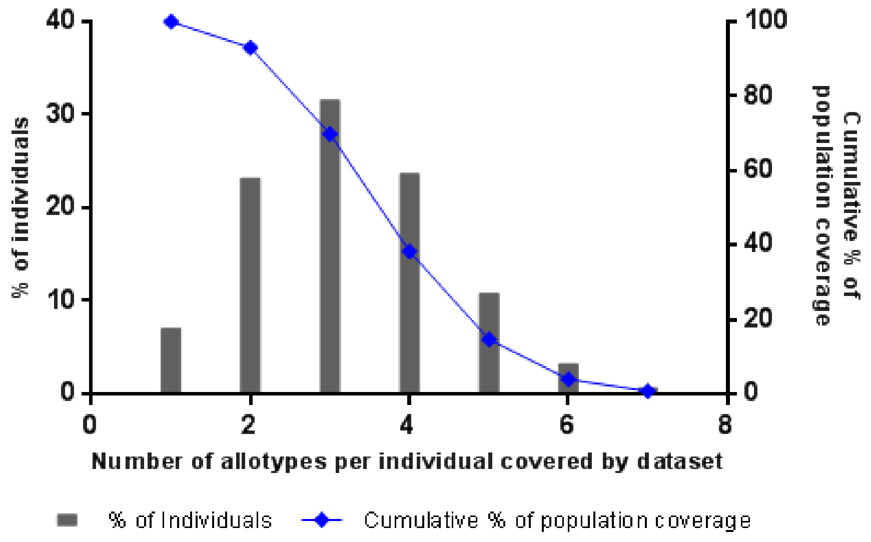

Supplemental Figure S1: **Coverage of the worldwide population.** Number of HLA allotypes covered per individual (grey) and cumulative percentage of individuals covered in the worldwide population (blue) considering the HLA class I and II alleles of THP-1.

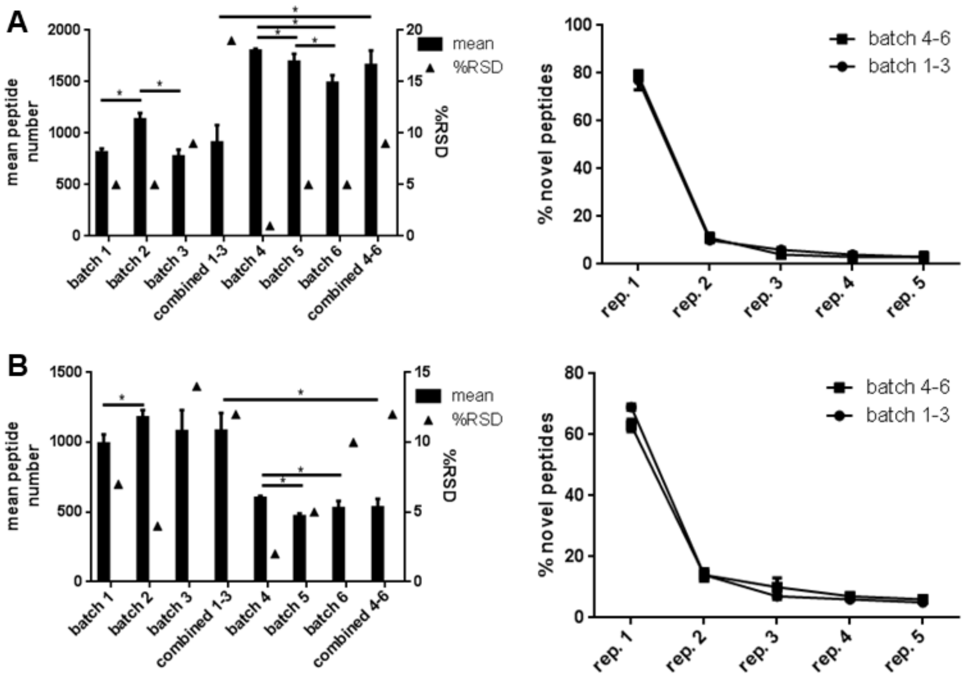

Supplemental Figure S2: **Reproducibility of peptide yields, deviations, and saturations.** Average peptide yields, deviations, and saturation with identified novel peptides of the identifiable sequences of untreated THP-1 cells after five technical LC-MS/MS replicates in the independently and jointly cultivated THP-1 populations batch 1-3 and 4-6 for HLA class I (A) and II (B).

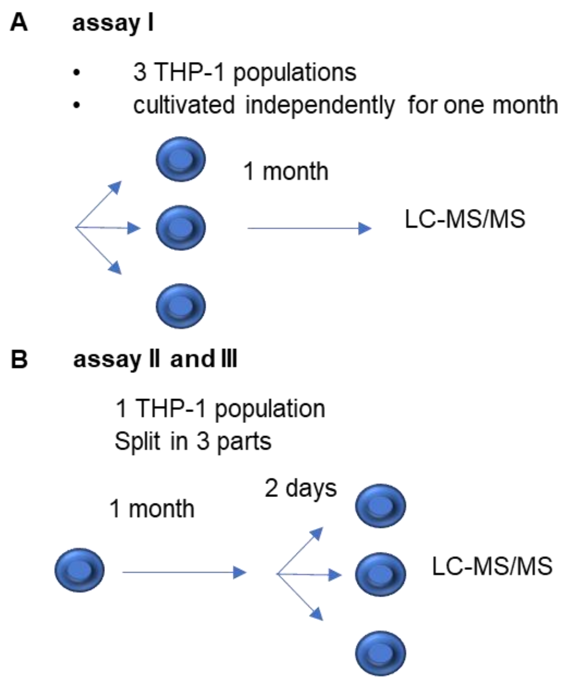

Supplemental Figure S3: Cultivation influence on the immunopeptidome. Cultivation scheme of THP-1 cells cultivated in three populations for one month with subsequent immunopeptidome analysis of each population (A, assay I) or cultivated in one population for one month and cultivated separately for two days prior to harvest and analysis (B, assay II and III). For material testing, materials were added on the last day of approach B for 24 h, until the cells were harvested for analysis.

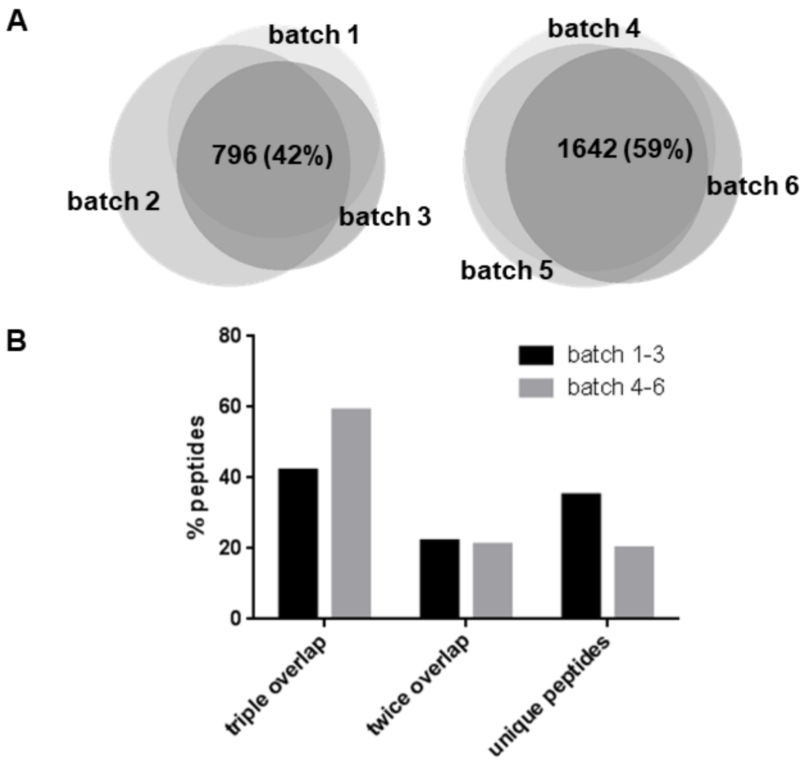

Supplemental Figure S4: Reproducibility of HLA class I peptide identifications. Venn diagrams with number and percentage of the peptide overlap of three biological replicates of independently (batch 1-3) and jointly cultivated (batch 4-6) THP-1 populations for HLA class I (A) and percentage of peptides present in all three, two or only one replicate (B).

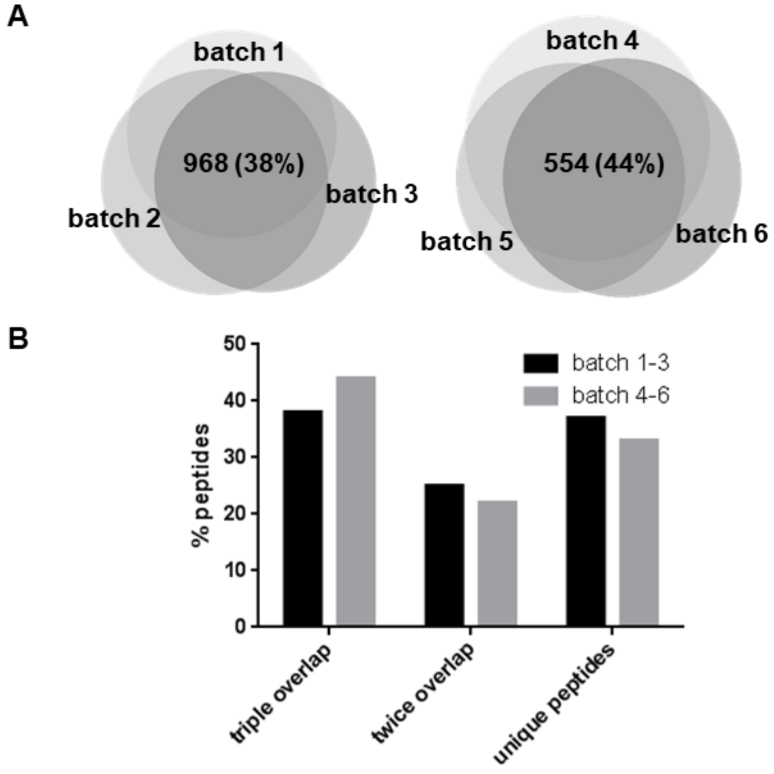

Supplemental Figure S5: Reproducibility of HLA class II peptide identifications. Venn diagrams with number and percentage of the peptide overlap of three biological replicates of independently (batch 1-3) and jointly cultivated (batch 4-6) THP-1 populations for HLA class II (A) and percentage of peptides present in all three, two or only one replicates (B).

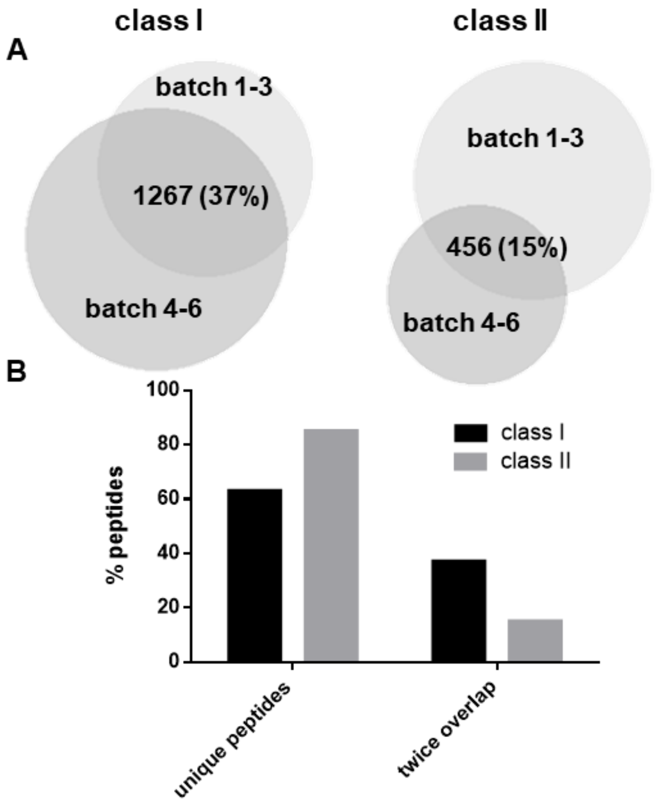

Supplemental Figure S6: Reproducibility of peptide overlap. Venn diagrams with number and percentage of the peptide overlap of independently cultivated (batch 1-3) and jointly cultivated (batch 4-6) THP-1 populations combined for HLA class I and II (A) and percentage of shared and unique peptides (B).

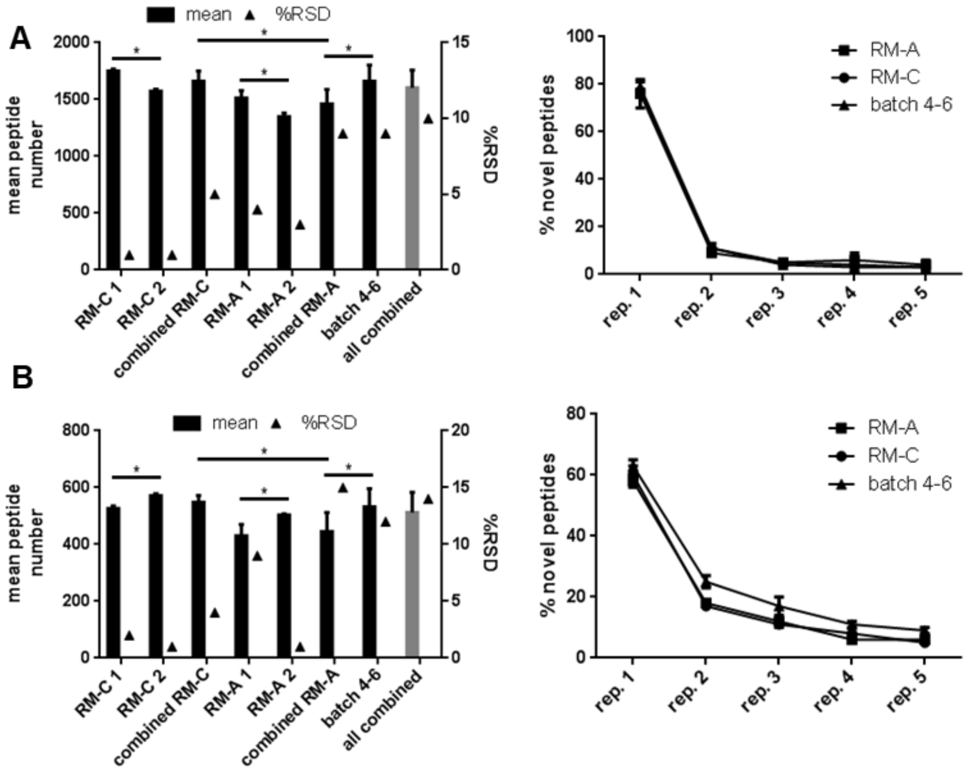

Supplemental Figure S7: Reproducibility of peptide yields, deviations, and saturations of material incubated samples. Average peptide yields, deviations, and saturation with identified novel peptides of the identifiable sequences of RM-A- and RM-C-incubated and untreated THP-1 cells (batch 4-6) after five technical LC-MS/MS replicates for HLA class I (A) and II (B).

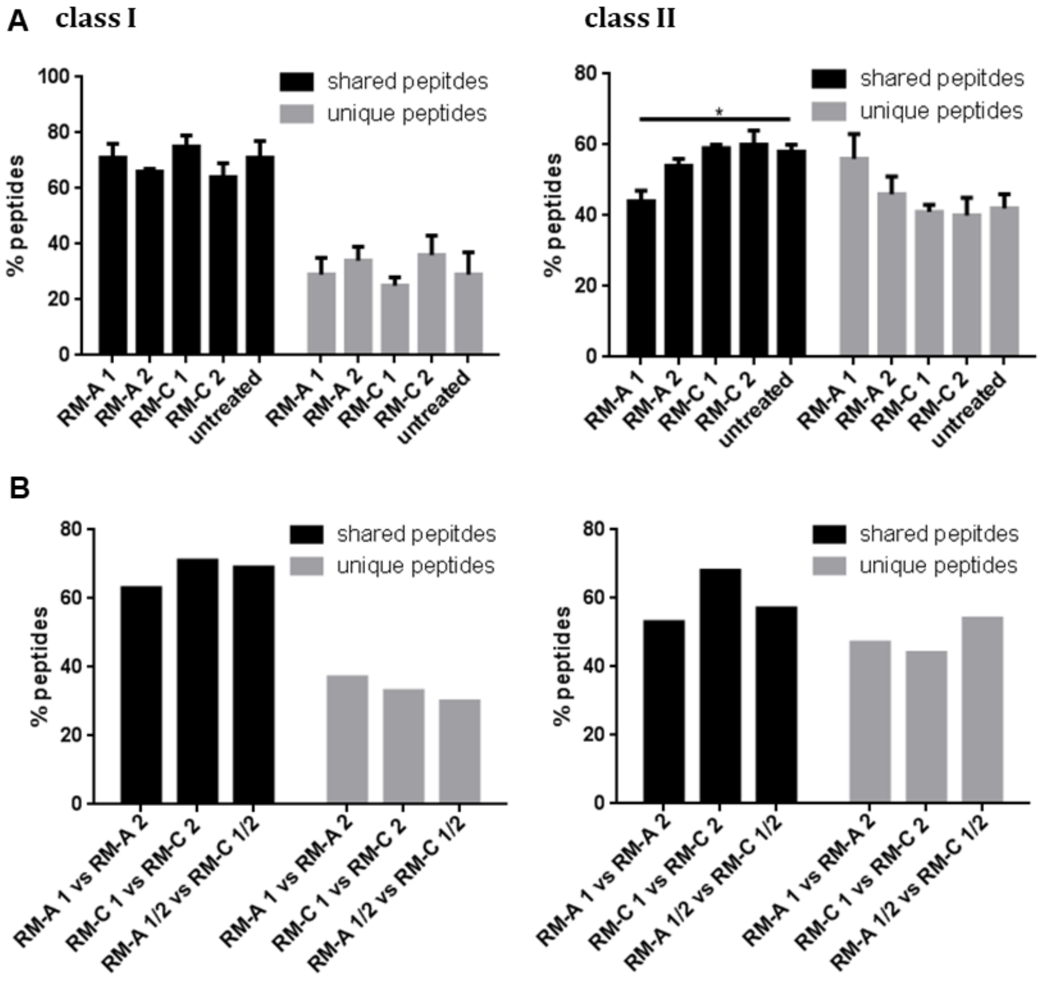

Supplemental Figure S8: Screening for material-exclusive peptides. Shared and unique peptides after overlapping of the peptides of each RM-A- (RM-A 1, 2) and RM-C-incubated sample (RM-C 1, 2) with each of the three untreated samples (batch 4-6) and the three untreated samples with each other (untreated), for HLA class I and II (A). Shared and unique peptides after overlapping of the exclusive peptides of each biological RM-A- and RM-C-incubated sample, after removing the peptides from batch 4-6 (B).

1.2.1 Supplemental Figure Legend

Supplemental Figure S9: Significantly modulated peptides. Volcano plots of the relative abundances of HLA class I- and II-presented peptides of THP-1 cells analyzed by label-free quantitation. Panels show in volcano plots part 1-6 all untreated and material and LPS incubated cells from assay I, II and III. Each spot corresponds to a specific HLA-presented peptide. Log2-fold changes in peptide abundance are displayed on the x-axis, corresponding significance levels after multi-test correction ($-\log_{10}$ *p*-value) on the y-axis. The number and percentage of peptides significant up- (red) or down-modulated (blue) ($\geq \log_2$ 2-fold change in frequency with $p < 0.01$) are indicated in the left and right top corner. Abbreviations: vs., versus; FC, fold-change.
