# Supplementary material for: The Impact of Biomaterial Cell Contact on the Immunopeptidome
Source: Front Bioeng Biotechnol. 2020 Dec 16;8:571294. doi: 10.3389/fbioe.2020.571294 (PMC7773052; doi:10.3389/fbioe.2020.571294)
Supplement: Supplementary file 1 [file Data_Sheet_1.zip › Supplemental Table S1.PDF]

Supplemental Table S1:

assay	stimulation	number of million cells	number of replicates	HLA class	total number of unique peptides	purity	raw file name
I	-	100	5	I	1205	99%	THP_class I_assayI_1_1-5
I	-	100	5	II	1562		THP_class II_assayI_1_1-5
I	-	100	5	I	1540	99%	THP_class I_assayI_2_1-5
I	-	100	5	II	1826		THP_class II_assayI_2_1-5
I	-	100	5	I	1152	99%	THP_class I_assayI_3_1-5
I	-	100	5	II	1740		THP_class II_assayI_3_1-5
II	-	100	5	I	2331	99%	THP_class I_assayII_1_1-5
II	-	100	5	II	979		THP_class II_assayII_1_1-5
II	-	100	5	I	2244	100%	THP_class I_assayII_2_1-5
II	-	100	5	II	856		THP_class II_assayII_2_1-5
II	-	100	5	I	2101	100%	THP_class I_assayII_3_1-5
II	-	100	5	II	923		THP_class II_assayII_3_1-5
II	RM-A	100	5	I	2165	99%	RMA_class I_assayII_1_1-5
II	RM-A	100	5	II	734		RMA_class II_assayII_1_1-5
II	RM-A	100	5	I	2029	99%	RMA_class I_assayII_2_1-5
II	RM-A	100	5	II	879		RMA_class II_assayII_2_1-5
II	RM-C	100	5	I	2365	100%	RMC_class I_assayII_1_1-5
II	RM-C	100	5	II	936		RMC_class II_assayII_1_1-5
II	RM-C	100	5	I	2141	100%	RMC_class I_assayII_2_1-5
II	RM-C	100	5	II	968		RMC_class II_assayII_2_1-5
II	zinc washer	100	5	I	2138	100%	zincW_class I_assayII_1-5
II	zinc washer	100	5	II	741		zincW_class II_assayII_1-5
III	-	400	3	I	2645	98%	THP_class I_assayIII_1-5
III	-	400	5	II	2854		THP_class II_assayIII_1-5
III	2 aluminum	400	5	I	2033	99%	2Alu_class I_assayIII_1-5
III	2 aluminum	400	5	II	2584		2Alu_class II_assayIII_1-5
III	4 aluminum	400	5	I	2151	98%	4Alu_class I_assayIII_1-5
III	4 aluminum	400	5	II	2727		4Alu_class II_assayIII_1-5
III	8 aluminum	400	5	I	2270	99%	8Alu_class I_assayIII_1-5
III	8 aluminum	400	5	II	3008		8Alu_class II_assayIII_1-5
III	steel	400	5	I	1989	97%	steel_class I_assayIII_1-5
III	steel	400	5	II	3099		steel_class II_assayIII_1-5
III	copper	400	5	I	1971	97%	copper_class I_assayIII_1-5
III	copper	400	5	II	3070		copper_class II_assayIII_1-5
III	LPS	400	5	I	2403	98%	LPS_class I_assayIII_1-5

III	LPS	400	5	II	3009		LPS_class II_assayIII_1-5
III	Zinc sulfate	400	5	I	1734	97%	zincS_class I_assayIII_1-5
III	Zinc sulfate	400	5	II	2358		zincS_class II_assayIII_1-5
