# Supplementary material for: The Impact of Biomaterial Cell Contact on the Immunopeptidome
Source: Front Bioeng Biotechnol. 2020 Dec 16;8:571294. doi: 10.3389/fbioe.2020.571294 (PMC7773052; doi:10.3389/fbioe.2020.571294)
Supplement: Supplementary file 1 [file Data_Sheet_1.zip › Supplemental Table S4.PDF]

Supplemental Table S4

assay	sample	IL-4	IL-8	IL-10	GM-CSF	IFN-γ	MCP-1	MIP-1β	TNF-α	IL-1b	IL-1ra	IL-12p70	VEGF
	RM-C												
II	RM-A												
	zinc washer												
	copper		+										
	2 aluminum												
	4 aluminum												
III	8 aluminum												
	stainless steel												
	zinc sulfate												-
	LPS	+	+		+++	+++	+++	++++	+++	++			
