# Supplementary material for: The Impact of Biomaterial Cell Contact on the Immunopeptidome
Source: Front Bioeng Biotechnol. 2020 Dec 16;8:571294. doi: 10.3389/fbioe.2020.571294 (PMC7773052; doi:10.3389/fbioe.2020.571294)
Supplement: Supplementary file 1 [file Data_Sheet_1.zip › Supplemental Table S5.PDF]

Supplemental Table S5

A

assay	up-modulated	aluminum	copper	LPS	steel	zinc sulphate	RM-A	RM-C	zinc washer
III	aluminum		29%	76%	53%	35%			
	copper	45%		45%	45%	45%			
	LPS	46%	18%		29%	39%			
	steel	53%	29%	47%		24%			
	zinc sulphate	16%	14%	30%	11%				
II	RM-A							63%	28%
	RM-C						26%		12%
	zinc washer						24%	24%	

assay	down-modulated	aluminum	copper	LPS	steel	zinc sulphate	RM-A	RM-C	zinc washer
III	aluminum		71%	60%	80%	53%			
	copper	58%		55%	65%	55%			
	LPS	66%	73%		76%	54%			
	steel	63%	63%	54%		56%			
	zinc sulphate	46%	58%	42%	62%				
II	RM-A							46%	42%
	RM-C						55%		15%
	zinc washer						73%	22%	

B

assay	up-modulated	aluminum	copper	LPS	steel	zinc sulphate	RM-A	RM-C	zinc washer
III	aluminum		68%	72%	77%	35%			
	copper	20%		53%	52%	22%			
	LPS	21%	55%		57%	22%			
	steel	18%	43%	45%		19%			
	zinc sulphate	13%	29%	27%	30%				
II	RM-A							30%	43%
	RM-C						33%		14%
	zinc washer						60%	18%	

assay	down-modulated	aluminum	copper	LPS	steel	zinc sulphate	RM-A	RM-C	zinc washer
III	aluminum		32%	25%	56%	80%			
	copper	18%		38%	63%	75%			
	LPS	19%	52%		61%	60%			
	steel	23%	46%	32%		63%			
	zinc sulphate	15%	25%	15%	29%				
II	RM-A							28%	72%
	RM-C						69%		66%
	zinc washer						77%	29%	
