# Supplementary material for: The Impact of Biomaterial Cell Contact on the Immunopeptidome
Source: Front Bioeng Biotechnol. 2020 Dec 16;8:571294. doi: 10.3389/fbioe.2020.571294 (PMC7773052; doi:10.3389/fbioe.2020.571294)
Supplement: Supplementary file 1 [file Data_Sheet_1.zip › Supplemental Table S6.PDF]

# Supplemental Table S6

| | group | protein/ gene | sequence | aluminum | copper | LPS | steel | zinc sulphate | RM-A | RM-C | zinc washer |
|---|---|---|---|---|---|---|---|---|---|---|---|
| increased | fibrosis | PSMA5 | KLNATNIEL | ✓ | ✓ | ✓ | | ✓ | | | |
| | autoantigen | Dystonin | IVSKSPVQF | | ✓ | ✓ | | | | | |
| | | | IQGSELPEL | | | | | | ✓ | ✓ | |
| | | | FIWENIHTL | | | | | | | | ✓ |
| | cytotoxicity | UNC13D | HPITSIEY | | | ✓ | | | | ✓ | |
| | inflam. resp. | CGAS | RWFQPAIPSW | | | | | | | | ✓ |
| | | | VPRIQLEEY | ✓ | ✓ | ✓ | ✓ | ✓ | | | |
| | stress resp. | SYVN1 | NPWDNKAVY | ✓ | ✓ | ✓ | ✓ | ✓ | | | ✓ |
| decreased | fibrosis | PSMD6 | MDYKTFVTY | ✓ | ✓ | ✓ | ✓ | ✓ | | | |
| | | | RYSVFFQSL | | | | ✓ | ✓ | | ✓ | |
| | autoantigen | TRIM21 | VPLEEAAQEY | ✓ | ✓ | ✓ | ✓ | ✓ | | | |
| | cytotoxicity | PUM3 | EAIREAVVY | ✓ | ✓ | ✓ | ✓ | ✓ | | | |
| | inflam. resp. | PIK3CD | GLTPHLTMV | | ✓ | | | ✓ | ✓ | | ✓ |
| | | | QYEPLFHML | ✓ | ✓ | ✓ | ✓ | ✓ | | | |
| | stress resp. | TMBIM6 | YLFLGGILM | | | | | | ✓ | ✓ | ✓ |
| | | | YVHMVTHF | ✓ | ✓ | ✓ | ✓ | ✓ | | | |
