# Supplementary material for: The Impact of Biomaterial Cell Contact on the Immunopeptidome
Source: Front Bioeng Biotechnol. 2020 Dec 16;8:571294. doi: 10.3389/fbioe.2020.571294 (PMC7773052; doi:10.3389/fbioe.2020.571294)
Supplement: Supplementary file 1 [file Data_Sheet_1.zip › Supplemental Table S7.PDF]

Supplemental Table S7

	group	protein/ gene	sequence	aluminum	copper	LPS	steel	zinc sulphate	RM-A	RM-C	zinc washer
increased	fibrosis	NHP2	EEVQSLPLPL				✓				
			EVQSLPLPL		✓	✓	✓				
			LEEVQSLPLPL		✓		✓				
	autoantigen	CALR	DLWQVKSGTIFDNF	✓		✓	✓	✓			
			GPGTKKVHVIFNYKGKNVLIN					✓			
			GVLGLDLWQVK					✓			
			LDLWQVKSGTIFDNF		✓	✓	✓	✓			
			WQVKSGTIFDNF	✓	✓	✓	✓	✓			
	cytotoxicity	SRGN	GPMFELLPGESNKIP					✓			
			LLPGESNK		✓	✓					
	inflam. resp./ stress resp.	GAPDH	AAFNSGKVDIVAINDPFIDL	✓	✓	✓	✓				
			ISWYDNEFGYSNRVVDL		✓	✓	✓	✓			
			ISWYDNEFGYSNRVVDLMAHMASKE		✓	✓	✓	✓			
			MAHMASKE		✓						
			NSGKVDIVAINDPFIDL		✓	✓					
			SDTHSSTFDAGAGIALNDHFV							✓	
			VDIVAINDPFIDL		✓		✓	✓			
			WYDNEFGYSNRVVDLMAHMASKE		✓	✓					
decreased	fibrosis	RPS27A	KNKHKRKKVKL						✓		
			TTPKKNKHKRKKVKL						✓	✓	✓
	autoantigen	CALR	KVHVIFNYKGKNVLI	✓		✓		✓			
			SPDPSIYAYDNFGVL		✓	✓	✓				
			SPDPSIYAYDNFGVLG				✓				
			SPDPSIYAYDNFGVLGLD		✓	✓	✓				
	cytotoxicity/ inflam. resp./ stress resp.	APP	DDLQPWHSFGADS						✓		✓
			DDLQPWHSFGADSVPA						✓		
			DDLQPWHSFGADSVPAN					✓			
			DDLQPWHSFGADSVPANT					✓			
			EFSLDDLQPWHSFGADSVPAN		✓			✓			
			EFSLDDLQPWHSFGADSVPANT					✓			
			GEFSLDDLQPWHSFG							✓	✓
			GEFSLDDLQPWHSFGAD		✓	✓	✓	✓			
			GEFSLDDLQPWHSFGADSVPA		✓	✓	✓	✓			
			GEFSLDDLQPWHSFGADSVPAN	✓	✓	✓	✓	✓			
			LDDLQPWHSFGADS						✓		
			LDDLQPWHSFGADSVPAN						✓		✓
			LDDLQPWHSFGADSVPANTENE					✓			
			MDAEFRHDSGYEVHHQK		✓	✓		✓			
			NGEFSLDDLQPWHSFGADSVPA		✓	✓	✓	✓			
			SLDDLQPWHSFGAD					✓			
			SLDDLQPWHSFGADSVPAN								✓
