# Supplementary material for: The Impact of Biomaterial Cell Contact on the Immunopeptidome
Source: Front Bioeng Biotechnol. 2020 Dec 16;8:571294. doi: 10.3389/fbioe.2020.571294 (PMC7773052; doi:10.3389/fbioe.2020.571294)
Supplement: Supplementary file 1 [file Data_Sheet_1.zip › Supplemental Table S8.PDF]

Supplemental Table S8

class I	shared aluminum samples	% of total peptides	class II	shared aluminum samples	% of total peptides
up-modulated	2+4	36% (n=66)	up-modulated	2+4	9% (n=29)
	4+8	34% (n=34)		4+8	22% (n=71)
	2+4+8	24% (n=44)		2+4+8	7% (n=23)
	2+4 or 4+8 or 2+8	54% (n=98)		2+4 or 4+8 or 2+8	30% (n=74)
down-modulated	2+4	40% (n=120)	down-modulated	2+4	18% (n=38)
	4+8	48% (n=144)		4+8	14% (n=14)
	2+4+8	32% (n=95)		2+4+8	10% (n=21)
	2+4 or 4+8 or 2+8	60% (n=177)		2+4 or 4+8 or 2+8	29% (n=40)
