# Supplementary material for: The Impact of Biomaterial Cell Contact on the Immunopeptidome
Source: Front Bioeng Biotechnol. 2020 Dec 16;8:571294. doi: 10.3389/fbioe.2020.571294 (PMC7773052; doi:10.3389/fbioe.2020.571294)
Supplement: Supplementary file 1 [file Data_Sheet_1.zip › Supplemental Table S9.PDF]

Supplemental Table S9

HLA class	modulation	slope	dose responsive peptides	aluminum treatment 2	4	8	UniProt accession	material associated function
I	increased	-	-	-	-	-	-	-
	decreased	Increasing > 0.5 fc/cond	GPLEVAQVF	-6,9	-3,1	-2,2	DOCK6; DOCK7; DOCK8	N/A
		Decreasing < -0.5 fc/cond	DTYmKASAPY	-2,5	-8,3	-8,9	CPSF7	
		Decreasing < -0.5 fc/cond	SPYNEVLSF	-4,4	-7,4	-7,9	ZMPSTE24	**M, IR, SR, A**
		Decreasing < -0.5 fc/cond	YVHMVTHF	-2,4	-3,7	-5,6	TMBIM6	**A, M, SR**
II	increased	Increasing > 0.5 fc/cond	SHYEEGPGKNLPFSVENKWSLL	6,4	7,0	8,0	COX7C	
		Increasing > 0.5 fc/cond	TVGGTAYAIYELA	2,1	3,3	4,2	COX7A2	
		Increasing > 0.5 fc/cond	LAVASFPKKQE	2,4	2,9	3,7	COX7A2	
	decreased	Increasing > 0.5 fc/cond	SGRTTGIVMDS	-4,7	-3,9	-2,3	ACTBL2; POTEKP; ACTB; ACTG1; POTEE; POTEF; POTEI; POTEJ	N/A
		Decreasing < -0.5 fc/cond	IDKDKPKEAVTVAVKMLKDDATEKD	-3,5	-7,4	-8,8	FGFR2	**A, D, WH**
